# A Comprehensive Review on the Biological, Agricultural and Pharmaceutical Properties of Secondary Metabolites Based-Plant Origin

**DOI:** 10.3390/ijms24043266

**Published:** 2023-02-07

**Authors:** Hazem S. Elshafie, Ippolito Camele, Amira A. Mohamed

**Affiliations:** 1 School of Agricultural, Forestry, Food and Environmental Sciences, University of Basilicata, 85100 Potenza, Italy; 2 Department of Basic Science, Zagazig Higher Institute of Engineering and Technology, 44519 Zagazig, Egypt

**Keywords:** natural products, metabolism, metabolomics, biological activity, plant diseases, crop protection

## Abstract

Natural products are compounds produced by living organisms and can be divided into two main categories: primary (PMs) and secondary metabolites (SMs). Plant PMs are crucial for plant growth and reproduction since they are directly involved in living cell processes, whereas plant SMs are organic substances directly involved in plant defense and resistance. SMs are divided into three main groups: terpenoids, phenolics and nitrogen-containing compounds. The SMs contain a variety of biological capabilities that can be used as flavoring agents, food additives, plant-disease control, strengthen plant defenses against herbivores and, additionally, it can help plant cells to be better adapted to the physiological stress response. The current review is mainly focusing on certain key elements related to the significance, biosynthesis, classification, biochemical characterization and medical/pharmaceutical uses of the major categories of plant SMs. In addition, the usefulness of SMs in controlling plant diseases, boosting plant resistance and as potential natural, safe, eco-friendly substitutes for chemosynthetic pesticides were also reported in this review.

## 1. Introduction

Natural products (NPs) are chemical substances isolated from living organisms and can be originated from microbial, plant or animal origins [1,2]. In addition, NPs are synthesized through the primary and secondary metabolism pathways. Metabolism is the sum of the chemical reactions that take place within cells of living organisms to provide energy for vital processes such as growth, development, reproduction, environmental adaptation and synthesizing new organic material [3]. Plants-based metabolites, widely used in agricultural, medical and pharmaceutical products, are divided into two main categories: (i) primary metabolites (PMs) and (ii) secondary metabolites (SMs).

The PMs include carbohydrates, proteins, fats and alcohol, which are involved in growth, development and reproduction [4,5,6]. Plant PMs are produced during the growth phase of the organism called trophophase at a high rate in the body [7,8], where they are constantly required for several essential processes of the body.

Unlike the PMs, the absence of SMs does not lead to direct cell death, but it can adversely affect the organism’s survivability in the long term. The SMs are often restricted to a narrow set of species within a phylogenetic group [9]. Plant SMs is the generic term used for more than 12,000 alkaloids, 40,000 terpenoids and 8000 phenyl propanoids, which are exclusively produced by plants. However, many terpenoids are at the interface between PMs and SMs. Furthermore, SMs can be used as a taxonomic character for the plant classification, where they can differentiate between families, genus and species [10,11]. The SMs are directly involved in plant defense, systemic resistance and other ecological functions [12] and are subdivided into three major classes: terpenoids, phenolic compounds and nitrogen-containing compounds [13,14,15,16]. Among the uses of SMs, they can carry out many protective functions in the human body as enhancing the immunity system against several pathogenic microbes. The biosynthesis of SMs is derived from primary metabolism pathways, which include the tricarboxylic acid cycle (TCA), methylerythritol-4-phosphate (MEP) pathway and the mevalonic and shikimic acid pathway. On the other hand, the phenylpropanoid pathway is also considered a rich source of plant metabolites, which are principally required for the biosynthesis of lignin and many other important compounds, such as flavonoids, coumarins and lignans.

## 2. Natural Products—Historical Overview

For thousands of years, nature has provided medicinal agents, and an astounding number of modern medications have been developed from natural sources, many of which are used in traditional medicine [17]. Natural products commonly refer to herbal concoctions, dietary supplements, and traditional and alternative medicines [18]. Plants have been utilized for medicinal purposes since the ancient Sumerian population. Hippocrates used about 400 distinct plant species for medicinal purposes [18]. Particularly, NPs have been used since ancient times and civilizations, including Chinese, Ayurveda and Egyptian, for the treatment of many diseases and illnesses [3]. The vast majority of traditional institutions during the Middle Ages in Europe, such as the classical Greek, Roman and Arabic regions, had their own herbal gardens for healing patients and teaching purposes about herbal plants. About 75 percent of people worldwide still rely on plant-based traditional medicines for primary health care [19].

During the last century, the term ’secondary metabolites’ was first suggested by Albrecht Kossel in 1910 and then by a Polish botanist Friedrich Czapek, 30 years later, who described SMs as the end products of nitrogen metabolism [20,21]. The most historically famous NP is penicillin which is derived from the fungus *Penicillium notatum* and was discovered by Fleming in 1929 [22,23]. The interesting medicinal uses of plant NPs have been well-documented for thousands of years. Among the most important plant NPs are SMs which have been used as an important source of medicines for early drug discovery [24,25]. The development of significant sophisticated analytical techniques, such as chromatography, in the middle of the 20th century made it possible to learn more about SMs and their chemical structures. Paper chromatography, in particular, made it clear that some of these compounds were colored pigments. Molecular biology and the significant advancement of biochemical tools in recent years have shown that plant SMs are crucial for plants to their environment adaptation [21].

For the comprehension of their distribution and abundance, plant SMs were considered not only as plant waste products but as specialized single substances with specific characteristics that originated from different chemical functional groups, and they can be useful in the pharmaceutical industry for discovering new bio-drugs for different purposes. Attempts to create models of allocation to SMs were immediately met with two difficulties: (i) comprehending the development and upkeep of the astonishing diversity of chemicals produced in the plant world; (ii) identifying the patterns underlying the variance in quantities that may be seen in both inside and between plants of the same and different species.

## 3. Uses of Plant Natural Products

The plant NPs and their semisynthetic derivatives are the richest sources of biologically active compounds [26]. Primary metabolites (PMs) such as carbohydrates, amino acids, fatty acids and organic acids are involved in growth and development, respiration and photosynthesis, and hormone and protein synthesis [4,12]. Plant secondary metabolites (SMs) such as terpenoids, phenolics and nitrogen-containing compounds (alkaloids) determine the color of vegetables, protect plants against herbivores and microorganisms and act as signal molecules under stress conditions [7,16]. However, most alkaloids are usually colorless crystals and do not have an effect on the color of vegetables, and some alkaloids are colored, such as berberine (yellow) and sanguinarine (orange) [7,16]. Plant SMs have several benefits of being utilized in different fields such as nutritional, cosmetic, medical, pharmaceutical and agricultural.

On the other hand, there are promising efforts have recently been conducted in order to increase the discovery of plant SMs and their uses, focusing mainly on the following areas: (i) increasing the productivity of target single natural substance through the cell culturing process by altering the processing and bioreactor performance, or by using elicitors and/or other techniques, regardless of their underlying processes [27]; (ii) establishing the possible signaling pathways involving directly or indirectly the creation of some target SMs; (iii) investigating genetically the methods of regulating some transcription factors, such as altering the regulator genes to enhance the biosynthesis of target and desired SMs [28,29]; (iv) measuring and evaluating global gene expression under various settings to examine the gene transcripts for plant SMs in order to understand their mechanism of action better [30]. A detailed illustration of the various applications for plant SMs has been reported as follows.

### 3.1. Nutritional Uses and Food Industry

Recently, there has been a great interest in the benefits of a wide variety of fruits and vegetables, which provide different bioactive SMs, including phytochemicals, vitamins, essential amino acids, minerals and fibers. However, the stability of those substances can be adversely influenced by various ecological and physical factors such as light, temperature and relative humidity [31]. On the other hand, the essential fatty acids extracted from several fruits and seeds oils have functional properties for reducing disease risk.

Some essential amino acids (EAA), such as histidine, isoleucine, leucine, lysine, methionine, phenylalanine, threonine, tryptophan and valine, are very important for diet and for several bodily processes, such as protein synthesis, tissue repair and nutrient absorption [32]. Some EAAs can enhance post-operative recovery; improve mood, sleep and athletic performance; and prevent muscle loss [32]. The majority of people may achieve their daily demands by eating a healthy, balanced diet containing EAA. In addition, the use of a diet rich in fiber has been recommended as a nutritional supplement against hypertension, obesity and diabetes [31].

Among the main functions of plant metabolites, they can influence the physical food characteristics such as color, taste, consistency and smell [33]. In addition, they have several health benefits; for example, they are characterized by antioxidant, anti-carcinogenic, anti-inflammatory and antimicrobial properties. Many plant metabolites are also able to lower the cholesterol level of humans and improve poor nutrition [34]. For example, the β-carotene (provitamin A) content in golden rice is significantly improved, which is helpful in fighting against vitamin A deficiency [34]. Many other phytochemicals are also regarded as nutrients that benefit human health. These include glucosinolates, flavonoids, phytosterols, phenolic acids, carotenoids and polyunsaturated fatty acids, which are efficient at reducing the chance of developing clinical disease [35,36].

### 3.2. Cosmetic Uses

The use of some plant SMs in cosmetic preparations is principally due to their low mammalian toxicity [37]. They are used for skin care, such as dryness, eczema, acne, free-radical scavenging and other skin protection effects. In addition, they are also used as hair growth stimulants and hair colorants. Among the most important plant SMs the essential oils (EOs), which can be incorporated into some cosmetic products giving a pleasant aroma, shine and other conditioning properties [38]. For example, menthol is often used as a flavoring agent in toothpaste and mouthwashes, which combines antibacterial efficacy and protects the mouth, and the addition of menthol gives a great-tasting freshness and prevents the buildup of plaque that can lead to gingivitis and bad breath [39]. Traditional remedies based-natural products have been used for centuries for treating skin and a wide variety of dermatological disorders [40]. In several plant EOs, the hydrophobic liquid mixtures of volatile compounds, such as castor, coconut, sunflower and olive oils, have been used for cosmetic purposes such as dry skin, eczema, acne and spot treatments [41]. Plant EOs are also known as volatile oils, ethereal oils, aetheroleum or simply as plant oil from which they were extracted.

### 3.3. Medical/Pharmaceutical Uses

Plant SMs and their derivatives have been employed as therapeutic agents for the treatment of numerous diseases and illnesses since ancient times [42]. Many natural compounds based-plant origin have been utilized as the main raw materials for several drugs. Plant SMs have also been used as drug precursors, prototypes and pharmacological probes [42,43]. In particular, a reasonable percentage of drugs worldwide are based on plant origin, and several bioactive compounds are currently used in the pharmaceutical industry [44].

Some specific examples of plant SMs have been used as drug precursors, such as morphine, the first NPs isolated from the opium poppy (*Papaver somniferum*) in 1806 and used as drug precursors for several medicines [45]. Artemisinin isolated from *Artemisia annua* has been used as an antimalarial drug containing sesquiterpene lactone, which treats malignant cerebral malaria caused by *Plasmodium falciparum* [46]. Paclitaxel, isolated from *Taxus brevifolia*, a highly oxygenated tetracyclic diterpenoid, acts as an antimitotic agent that inhibits the polymerization of tubulin to form microtubules. The same substance has also been used as an effective drug against ovarian and breast cancers [47]. In addition, calanolide A, isolated from *Calophyllum lanigerum*, is a non-nucleoside reverse transcriptase inhibitor (NNRTi) of type-1 HIV and an inhibitor of AZT-resistant strains of HIV [48].

In addition, scientific research regarding the plant SMs is continuing to explore their chemical structures as possible templates for new drug developments [49]. On the other hand, the search for more effective anti-cytotoxic agents is still a priority in the development of new anticancer drugs. Therefore, a number of important commercialized novel anticancer treatments have been derived from plants. In particular, many plant SMs, including alkaloid, diterpenes, triterpenes and polyphenolic compounds, have already been reported as great anticancer agents in several research [50,51].

### 3.4. Agricultural Uses

The NPs can be used for plant protection and growth promotion. In fact, several botanical and microbial plant SMs may induce the plant systematic resistance and plant hormones, which positively enhance plant growth [52,53]. The literature reports that many plants, such as neem, citrus, sage, menth, garlic, oregano, moringa, etc., or their single bioactive constituents, have been used for controlling several bacterial and fungal diseases, pest infestation and harmful weeds [54,55,56]. The main applications of plant SMs for plant protection and as plant-growth promoters have been discussed as follows.

#### 3.4.1. Plant Protection

Plant NPs have several benefits for agriculture, where they can exhibit antiviral, antibacterial and antifungal activities [57]. NPs have economic importance; in fact, they can be used directly as active ingredients in several commercial products used for crop protection against phytopathogens, insects, weeds and mites. Many plant SMs, such as flavones, flavonoids, quinines, tannins, terpenes and saponins, act as antifungal and/or antibacterial agents for plant diseases [58]. In particular, some common bioactive SMs used for plant protection have very complex structures with many stereo centers, such as flavanones extracted from several citrus plants, has antimutagenic and antibacterial effects [59]. Azadirachtin, extracted from *Azadirachta indica*, has a strong insecticidal effect. Chelerythrine, extracted from Chelidonium majus, showed an important antibacterial effect. On the other hand, a lot of vegetal extract and plant EOs have been commercialized as crop protection products for use in biological agriculture [55,59,60,61,62]. In particular, some plant SMs, especially root exudates, play an important role in plant protection against a wide range of phytopathogens by killing or deterring soil microbes, herbivorous insects and nematodes [5,63,64]. A recent study conducted by Ensley [64] reported that pyrethrin derived from the flowers of *Pyrethrum cinerariaefolium* explicated promising insecticidal effects against some harmful pests.

#### 3.4.2. Plant Growth-Promoting Effect

Plant growth regulators are simple molecules that have important effects on plant growth and are effective even in low concentrations [65,66]. Many natural substances and their synthetic or semisynthetic derivatives have been used in agriculture as plant growth regulators or other essential processes such as germination, vegetative reproduction, maturation and senescence [67]. In particular, plant hormones, also defined as phytohormones, are chemical substances produced by plants that play an essential role in regulating the living plant functions such as growth (auxin indole-3-acetic acid; IAA) and reproduction, seed and bud dormancy, organizing stomatal closure, and abiotic stress responses and seed ontogeny (abscisic acid). These substances, derived mainly from SMs, are responsible for the adaptation of plants to environmental conditions. The plant response to SMs growth regulators may differ according to plant species, age, variety, environment, stage of development, physiological and nutritional status, and hormonal balance [66,68,69].

Many other plant-derived compounds have growth-promoting characteristics and defense functions, such as karrikins, which promote seed germination and plant growth by simulating the signaling hormone strigolactone and aid in the growth of symbiotic arbuscular mycorrhizal fungi in the soil [70]. In addition, melatonin is also considered an important plant NPs as a hormone and is synthesized indirectly as an intermediate product of the shikimate pathway and has some physiological functions for plant growth, reproduction and defense against oxidative stress [71].

On the other hand, several phytohormones play an essential role in the ability of the plant to adapt and accommodate to different abiotic and biotic stresses such as drought, salinity and temperature [72]. The positive effect of plant metabolites on the plant response to unfavorable external factors may help the plants to maintain optimal growth and development [73]. Some common plant SMs, such as abscisic acid, salicylic acid, gibberellins, cytokinin, jasmonic acid and ethylene, play major roles in plant defense responses to biotic and abiotic stresses [74]. On the other hand, Smolander et al. [74] reported that the decomposition of cultivated soil might be influenced by some plant SMs, particularly terpenes and tannins, through increasing nitrogen immobilization and C/N cycling.

## 4. Classification of Plant Metabolites

Plant metabolites are frequently distributed differently among a few taxonomic groups in the plant kingdom. In particular, plant SMs are an exceptional source of drugs, food additives and fine chemicals. They also offer unique materials that are used in different fields. The chemical synthesis of those natural compounds or their possible derivatives can be carried out not only through a direct extraction from plants but also by using plant cell culture, which becomes a promising alternative for manufacturing metabolites that are challenging to obtain through chemical synthesis or plant extraction [30]. Recently, the correct explanation of ‘Plant SMs’ term is the ‘specialized plant metabolites’, which is due to the wide variety of natural compounds in plant species that are usually playing different overlapping biological functions for systemic resistance, defence and growth-promoting effects.

From the classification point of view, plant cells produce two types of metabolites: primary metabolites (PMs) are involved directly in plant growth and metabolisms, such as carbohydrates, lipids and proteins; secondary metabolites (SMs) are considered the end products of primary ones, but not involved in the metabolic activity, such as (alkaloids, phenolics, steroids, essential oils, tannins, etc. [75]. The latter act as defense specialized chemicals essential for plant health, growth and cell health [75]. The detailed information on each category is explained below.

### 4.1. Primary Metabolites

Primary metabolites are the main products of metabolic processes involved in different living functions required for life, such as cellular functions, growth and reproduction. Among the most common PMs are carbohydrates, lipids, proteins and nucleic acids [76,77]. Production of PMs occurs during the active growth phase (trophophase) and begins in the presence of required nutrients in a growth medium. Plant PMs are also involved in energy production, including respiratory and photosynthetic enzymes. PMs are also the main components of the basic cell structures, such as phospholipids for cell membranes, peptidoglycan and chitin for cell walls, and proteins for cytoskeletons [78]. In addition, DNA and RNA, which store and transmit genetic information, are composed of nucleic acid primary metabolites [79]. On the other hand, PMs perform their functions as signal molecules to trigger defense response by signal transduction and pathogen recognition processes [80]. The signaling molecules such as hormones and other growth factors are composed of peptides, biogenic amines, steroid hormones, auxins and gibberellins, etc. [81].

### 4.2. Secondary Metabolites

Plants SMs attracted the interest of many scientists from different research fields in plant biology and biotechnology. They studied, in vitro and in vivo, bioactive molecules extracted from several plants with the aim to isolate, analyze and biochemically characterize new phytochemicals and evaluate their biological activity and eventual toxicity. Plant SMs are classified based on their chemical structures into the following major classes: terpenoids, phenolics compounds and nitrogen-containing compounds [82].

#### 4.2.1. Terpenoids

Terpenes are the largest group of plant SMs derived chemically from 5-carbon isoprene units [83,84]. The terpenoids have an important defensive role in some important plant species such as polypodium, peppermint, lemon, basil, sage, corn, cotton and wild tobacco [84,85]. Terpenes are classified according to the number of the following isoprene units: (i) monoterpenes consist of two isoprene units and have the molecular formula C_10_H_16_; (ii) sesquiterpenes consist of three isoprene units and have the molecular formula C_15_H_24_; (iii) diterpenes are composed of four isoprene units and have the molecular formula C_20_H_32_; (iv) for sesterterpenes, terpenes have 25 carbons and five isoprene units; (v) triterpenes consist of six isoprene units and have the molecular formula C_30_H_48_; (vi) sesquarterpenes are composed of seven isoprene units and have the molecular formula C_35_H_56_; (vii) tetraterpenes contain eight isoprene units and have the molecular formula C_40_H_64_; (viii) polyterpenes consist of long chains of many isoprene units; (ix) norisoprenoids are characterized by the shortening of a chain or ring by the removal of a methylene group or substitution of one or more methyl side chains by hydrogen atoms.

In particular, monoterpenes consist of two isoprene units. They are important components of plant essential oils in the families of *Lamiaceae*, *Pinaceae*, *Rutaceae* and *Apiaceae*. Monoterpenes are classified into unsaturated hydrocarbons (e.g., limonene), alcohols (e.g., linalool), alcohol esters (e.g., linalyl acetate), aldehydes (e.g., citronellal) and ketones (e.g., carvone) [86]. Sesquiterpenes consist of three isoprene units. They are classified into three main groups: (i) acyclic (e.g., farnesol), (ii) monocyclic (e.g., bisabolol) and (iii) bicyclic (e.g., caryophyllene) [87]. Diterpenes consist of four isoprene units. They are classified into acyclic and macrocyclic compounds. Diterpenoids, such as vitamin K1 and vitamin A, are important bioactive constituents of a number of medicinal plants [88]. Among the most important specialized metabolite within the terpenoids group are phytohormones such as gibberellins (GAs) diterpenes and abscisic acid (ABA) sesquiterpenes, which are considered two important phytohormones involved in various developmental processes, including tuberization with antagonistic [89]. GAs and ABA are essential regulators during stem/root tuber development [89].

**Figure 1:**
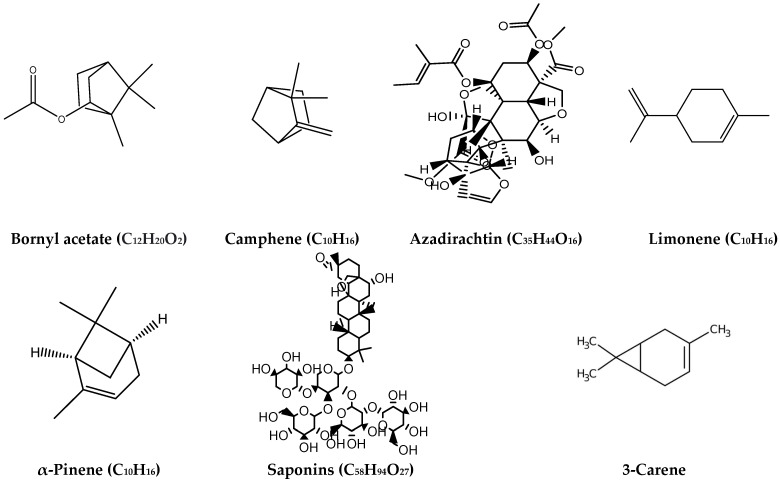
Chemical structures of some common plant terpenoids.

Table 1 illustrates some common important terpenoids. Azadirachtin, derived from seeds of the neem tree (*Azadirachta indica*), has an insecticidal activity; in fact, it is considered the main active ingredient in many pesticides such as treeAzin, azaMax, azaSol and terramera Cirkil [90]. Bornyl acetate (ester), one of the terpenoids present in many plant EOs, has a notable antibacterial activity [91]. Camphene (bicyclic monoterpene) is one of the most important terpenoids as a minor constituent of many aromatic EOs such as camphor, cymbopogon and ginger, whereas it is a major constituent of other EOs such as thymus, oregano and sage. Camphene has antibacterial and anticancer activities and is used as a medication for pulmonary disease [92,93]. Limonene (cyclic momoterpene) is derived from many plant species, such as citrus fruit oil, which shows effective antibacterial and antifungal activities and can also be used in food preservation [94,95]. α-Pinene (unsaturated bicyclic monoterpene) is extracted from different coniferous trees such as *Pinus silvestris*, *Pinus taeda* and *Pinus ponderosa* and has important antimicrobial, antioxidant, anti-inflammatory, anti-carcinogenic and insecticidal activities [96,97,98]. Saponins (triterpene glycosides), present in *Allium* species, asparagus, oats, spinach, sugar beet, tea and sweet potato, are characterized by hypolipidemic properties. In addition, saponins can reduce cholesterol and lipoprotein levels and show anti-diabetic and cytotoxic activity [99,100]. 3-Carene (bicyclic monoterpene) occurs naturally in a variety of aromatic plants such as rosemary, citrus, basil, cedar, pine and cannabis and has antimicrobial effect against some food-borne pathogenic bacteria both Gram-positive (G+ve) and Gram-negative (G-ve). 3-Carene can be used as a food additive and flavoring agent in the food industry [101,102]. The chemical structure of the plant terpenoids mentioned above is reported in Figure 1.

#### 4.2.2. Phenolic Compounds

Phenolic compounds (PCs) are one of the most important groups of plant SMs. The common characteristic of PCs is the presence of one or more phenol groups [103]. The PCs are widespread in plants where it contributes significantly to the color, taste and flavor [104]. Some PCs are soluble in organic solvents, some in water, and others are insoluble polymers [4]. In addition, PCs might be biosynthesized from the shikimate metabolic pathway [105]. Several PCs have pharmacological activities, including anti-inflammatory, antioxidant and antihepatotoxic effects. In particular, PCs can be classified according to their structures into simple phenolics, tannins and flavonoids [106].

Flavonoids are the largest group of naturally occurring phenols [107]. Flavonoids may be divided into various classes according to the oxidation level of the central ring. Flavonoids are present mainly in higher plants and are involved in nitrogen fixation and flower pigmentation [108]. It is secreted by the roots and the aid of Rhizobia in the symbiotic-infection stage [109]. There are more than 5000 identified flavonoids extracted from various plants divided according to their chemical structures into flavanones, flavanonols, flavans, anthocyanidins and isoflavonoids [108]. On the other hand, syringic acid is a phenolic substance that is frequently present in fruits and vegetables and is produced through the shikimic acid pathway [110]. It demonstrates a variety of therapeutic uses for diabetes prevention [110]. The main subclasses of flavonoids are flavonols, flavones, flavanones, anthocyanidins and isoflavones [104]. Other flavonoid groups with minor components are dihydroflavonols, flavan-3,4-diols, coumarins, chalcones, dihydrochalcones and aurones [111].

Table 2 illustrates some common important flavonoids. Flavonols, derived from different plants or plant sources such as onion, red wine, olive oil, berries and grapefruit, showed antimutagenic activity, decreasing the mutagenic effects of some potentially harmful chemicals [112,113,114]. Flavonols also have some antiviral activity, disturbing and destabilizing the binding between the S protein and ACE2 receptor leading to the inhibition of virus entry and able to halt the activity of several enzymes involved in the replication of the virus [115].

**Figure 2:**
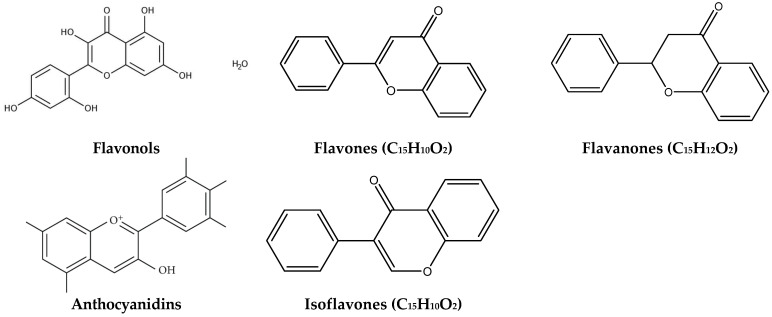
Chemical structures of some common plant flavonoids.

Flavones, derived from some fruit skins, red wine, buckwheat, red pepper and tomato skin, showed interesting antimutagenic activity and anti-inflammatory and antiviral effects [112,114]. Flavanones occur in different citrus fruits, grapefruits, lemons and oranges and show antimutagenic and antibacterial activities [112,114,116]. Flavanones, such as naringin and naringenin, have strong antioxidant effects and neuroprotection action for the central nervous system [117]. Anthocyanidins occur principally in cherry, raspberry and strawberry [113]. They are considered an important group of visible plant pigments with several health benefits against a variety of oxidant agents [117]. Isoflavones have antimutagenic and antibacterial activity and are largely produced by the members of the bean family, such as soybean [112,114]. Isoflavones can also be considered dietary supplements, but there are few studies regarding their health benefits. The chemical structure of the plant flavonoids mentioned above is reported in Figure 2.

**Table 2:** Some common plant flavonoids.

Class	Plant Source	Activity	Examples	References
Flavonols	Onion, red wine, olive oil, berries and grapefruit	Antimutagenic and antiviral	Quercetin, Kaempferol, Myricetin, Galangin, Isorhamnetin, Rhamnazin.	[112,113,114]
Flavones	Fruit skins, red wine, buckwheat, red pepper and tomato skin	Antimutagenic, antiviral and anti-inflammatory	Luteolin, Apigenin, Tangeritin.	[112,114]
Flavanones	Citrus fruits, grapefruits, lemons and oranges	Antimutagenic and antibacterial	Hesperetin, Naringenin, Eriodictyol, Homoeriodictyol.	[112,114,116]
Anthocyanidins	Cherry, raspberry and strawberry	Important plant pigments with several health benefits, such as protecting the body against a number of oxidant agents	Cyanidin, Delphinidin, Pelargonidin, Peonidin.	[113,118]
Isoflavones	Soybean	Antimutagenic and antibacterial	Daidzein, Genistein, Glycitein.	[112,114]

#### 4.2.3. Nitrogen-Containing Compounds

Nitrogen-containing compounds include alkaloids, cyanogenic glucoside and glucosinate [4]. Alkaloids, organic compounds containing nitrogen atoms, are naturally occurring in the plant kingdom. Alkaloids can be divided based on their chemical structures into heterocyclic and non-heterocyclic. Alkaloids are commonly present in some plant orders, such as *Caryophyllales, Magnoliales*, *Rosales* and *Gentiales* [107]. Alkaloids demonstrate different biological properties, such as pharmacological and cytotoxic effects for vertebrates and antimicrobial/antiviral properties [107,113].

Many alkaloids are valuable medicinal agents that can be utilized for treating various diseases, including malaria, diabetics, cancer, cardiac dysfunction, etc., such as quinine, ephedrine and homoharringtonine, which are used effectively for the treatment of malaria, inflammatory diseases and cancer therapy, respectively. Alkaloids play different roles for the plant during the metabolism process, such as storage reservoirs of nitrogen, protective agents against predators’ attacks and growth regulators. Among the alkaloids, cyanogenic glucosides are present in more than 2500 plant species and play an important role in plant defense against herbivores. It has a bitter taste and releases toxic hydrogen cyanide upon tissue disruption. Cyanogenic glycoside was present principally in the bitter almonds seeds and other fruits such as apricots, almonds, peaches and apples. It can also be present in wine; however, its content depends on the levels in respective fruits and other factors such as temperature and alcohol concentration [119].

Table 3 illustrates some common important alkaloids. Among them, morphine is one of the most important alkaloids derived from the poppy plant [120]. Morphine has analgesic and anesthetic activity and is also used to reduce anxiety [121]. Quinine derived from *Remijia* sp. has antimalarial activity [122]. Quinine was first isolated in 1820 from the bark of a cinchona tree native to Peru [122]. Quinine is included in the World Health Organization’s List of Essential Medicines for the treatment of malaria [123]. Ephedrine exists in *Ephedra* sp. and has anti-inflammatory activity [124], and it is used to prevent low blood pressure during anesthesia [125]. Homoharringtonine extracted from *Cephalotaxus fortuneiand* [126] was approved by the U.S. Food and Drug Administration (FDA) for the treatment of patients with chronic myeloid leukemia [127]. Homoharringtonine has been used in China for more than 50 years in the treatment of patients with myeloid leukemias. Homoharringtonine is considered an important anticancer agent even in cases of resistance to specific, targeted therapy such as tyrosine kinase inhibitors [127].

**Figure 3:**
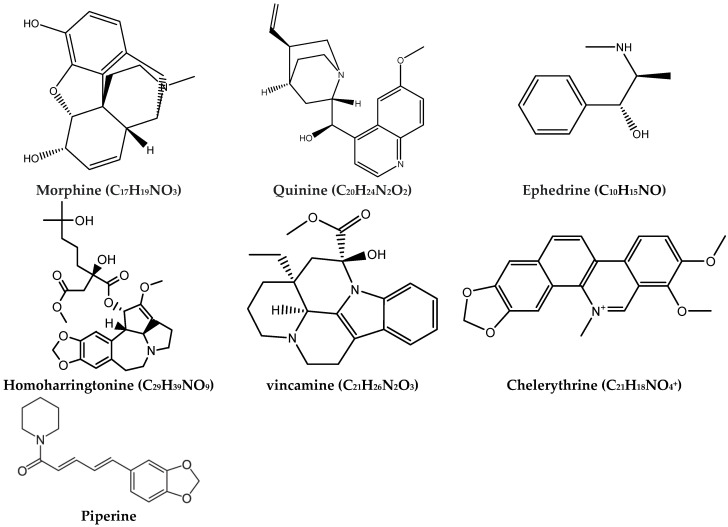
Chemical structures of some common plant alkaloids.

Vincamine extracted from *Vinca minor* play an important role as vasodilatory [128]. Vincamine is used for the treatment of primary degenerative and vascular dementia (in Europe) and as a dietary supplement in the USA [129]. Chelerythrine derived from *Chelidonium majus* has antibacterial activity [130,131]. This substance showed an anticancer effect and served as a base for many potential novel anticancer drugs. Piperine is extracted from fruits of *Piper longum* and *P. officinarum* and is used as an antihyperglycemic agent [132]. The chemical structure of the plant alkaloids mentioned above is reported in Figure 3.

## 5. Biological Activity of Plant SMs

For several centuries, the natural products based-plant origins, particularly plant SMs, have been effectively used in agriculture, the food industry and pharmacological fields for controlling different phytopathogens, destructive insects, harmful weeds, food preservation and pharmaceutical drugs for humans [133].

### 5.1. Antimicrobial

Among the most common plant SMs with antimicrobial effects against different pathogenic microorganisms are saponin, flavonoids, thiosulfinates, glucosinolates, phenolics, alkaloids and organic acids. The antimicrobial activity of plant SMs depends mainly on their chemical structures, principal constituents and effective dose. In particular, some terpenoids (aliphatic alcohols, aldehydes, ketones, acids) and some phenolic compounds (isoflavonoids) are considered the main plant components demonstrating efficient antimicrobial activity [134]. Shan et al. [135] reported that the antibacterial effect of more than 40 plant extracts is correlated to the presence of phenolic constituents. In addition, some vegetables, such as red cabbage, are rich in the phenolic compound anthocyanins, which showed promising antimicrobial activity [136]. On the other hand, some plant EOs containing phenolic compounds, such as citrus, olive, tea tree, orange and bergamot, have interesting antimicrobial activity against several pathogenic microbes. However, other researchers reported that some non-phenolic EOs extracted from oregano, clove, rosemary, parsley and sage showed promising antibacterial effects against both G+ve and G-ve pathogenic strains [51,61,137,138]. In addition, many plant oils are rich in phenolic compounds such as thymus, verbane, menth, lemon, orange, etc. [55,56,57,61,139,140]. In particular, some nonphenolic constituents of EOs, such as allyl garlic oil, are more effective, especially against G-ve bacteria [140,141].

### 5.2. Antioxidant

Antioxidants are compounds able to inhibit the oxidation process, the chemical process that can produce free radicals and chain reactions and may damage the cells. Antioxidants are useful for increasing the quality of processed food and avoiding spoilage [142]. The sources of antioxidants can be natural or synthetic. Certain fruits and vegetables in their organs are rich in antioxidants [18]. The plant SMs with potential antioxidant activity can be considered natural alternatives to many chemosynthetic substances for improving food quality and increasing self-life [143,144]. Several plant families, such as *Asteraceae*, *Rosaceae* and *Punicaceae*, produce bioactive SMs, including flavonoids, lignans, vitamins, carotenoids and terpenoid, which are characterized by strong antioxidant activities [145], as reported in Table 4. The natural antioxidants from plant materials are mainly polyphenols (phenolic acids, flavonoids, anthocyanins, lignans and catechin), carotenoids (xanthophylls, lycopene and carotenes) and vitamins (vitamins E and C).

In particular, pomegranate (*Punica granatum* L.) fruits in the family *Punicaceae* are rich in acids, sugars, vitamins, minerals and phenolic compounds as strong antioxidant agents [146]. Aqueous tea extract is one of the most common natural antioxidants used in the food industry due to its rich content of catechins, tannins and flavonoids without affecting the food flavor, as reported by Yin et al. [147]. In addition, broccoli, cabbage, tomatoes and lettuce produce some potential antioxidants, such as vitamins C and E, which aid in the solubility of lipid compounds and prevent cell damage originating from high oxidative stress [148]. On the other hand, several plant EOs extracted from oregano, marjoram, thyme, verbena, sage and rosemary are considered rich sources of natural antioxidants; however, their volatile nature affects the food flavor [149,150].

**Table 4:** Most common antioxidant-based plant origin.

Class	Plant Source	Function	Examples	References
Carotenoids	Carrot, pumpkins, winter squash, sweet potato	- Give the characteristic color to some vegetables (carrots, pumpkins corn, tomatoes) - Able to signal the production of absicisic acid, which regulates plant growth, seed dormancy, germination, cell division, stress responses)	Lycopene, alpha carotene, beta carotene, zeaxanthin.	[145,151]
Polyphenols	Berries, herbs and spices, cocoa, nuts, vegetables	Play diverse roles in the ecology of plants, such as releasing growth hormones such as auxin, prevention of microbial infections, Signaling molecules in ripening and other growth processes)	Flavonoids, tannic acid, ellagitannin, catechin.	[152,153]
Vitamin C	vegetables, berries, citrus fruits	Plays a role in controlling infections and healing wounds and is a powerful antioxidant.		[145,154]
Vitamin E	Oilseed, palm oil, nuts, eggs, dairy products, whole grains, vegetables, cereals, margarine, etc.	Used in skincare and wound-treatment products, but no clinical evidence.		[146,155]

### 5.3. Pharmacological Activity

#### 5.3.1. Antibiotic

Several plant SMs have antibiotic-action properties against a variety of harmful microbes interfering the essential cellular functions such as cell wall synthesis, DNA/RNA replication and protein assimilation. Between 1935 and 1968, 12 kinds of antibiotics were approved in the medicinal field [156]. However, from 1969 to 2000, their number significantly decreased. After that, the number of available antibiotics gradually expanded again between 2003 and 2015, when 20 new antibiotics were approved; among them, 16 were based on natural compounds and/or their derivatives [157].

Among the most recent common antibiotics from natural sources, we underline fidaxomicin (2010) and lipopeptide daptomycin (2003) derived from *Actinomycetes* and retapamulin (2007), a fungus-derived pleuromutilin extracted from *Pleurotus mutilus*, *Pleurotus passeckerianus* and *Clitopilus scyphoides* [156]. A recent study conducted by Newman and Cragg [158] reported that several plant SMs had been approved to possess interesting antibacterial activity, such as plazomicin and sisomicin, which were approved in the USA as two derivatives of the aminoglycoside. In addition, the same authors reported that some modifications of aminoglycosides were carried out in 2018, forming three tetracycline-based agents: omadacycline, eravacycline, sarecycline and lefamulin [158].

#### 5.3.2. Antiviral 

There is no efficient therapy against viruses because they use the biological mechanism of host cells for their replication; for this reason, all possible virus treatments have serious side effects on the host cells [159]. Natural compounds are considered important sources for the discovery and development of novel antiviral drugs due to their availability and minimal side effects. There are few drugs based plant sources available for controlling viral diseases. Recently, several research has been conducted to find out new plant sources serving as antiviral agents [160]. The world annual report of medicinal chemistry between 1983 and 1994 indicated that only seven natural drugs out of ten synthetic agents had been approved by Food & Drug Administration (FDA). Many natural and synthetic compounds showed in vitro antiviral activity with less effectiveness in vivo [159]. Most of the work related to antiviral compounds revolved around the inhibition of various enzymes associated with the life cycle of viruses.

Shu [161] reported that there are two effective plant-derived antiviral compounds, (+)-Calanolide A and SP-303, under clinical development. (+)-Calanolide A was isolated from the Malaysian rainforest tree *Calophyllum langigerum* and showed effective HIV-RT inhibitory activity [161]. SP-303 was isolated from the latex of a Latin American plant Croton lechleri, showing potential in vitro activity against Herpes simplex viruses (HSV) and other DNA or RNA viruses [161]. Several plant alkaloids such as Citrusinine I (isolated from *Citrus* sp.), Atropine (isolated from Atropa belladonna) and Scopolamine (isolated from *Datura stramonium*) illustrated antiviral activity against many viruses such as HSV [162,163]. However, some plant PMs such as carbohydrates, have also exhibited in vitro inhibitory activities against HIV, cytomegalovirus (CMV) and HSV, such as mannose (isolated from Cymbidium hybrid and Epipactis helleborine), show effective antiviral effects against HIV and CMV [162]. In particular, Shashank and Abhay [157] reported that the structure–function relationship between plant SMs and HIV enzyme inhibitory activity has also been observed.

#### 5.3.3. Anti-Inflammatory

Inflammation is a complex biological protective response of body tissues to microbial infection, tissue damage or irritants involving immune cells, blood vessels and molecular mediators [157]. Several studies reported the anti-inflammatory effects of some natural herbs, such as *Curcuma longa*, *Zingiber officinale*, *Rosmarinus officinalis* and *Borago officinalis*, which have promising applications in some clinical aspects [164]. Natural products recently developed as anti-inflammatory drugs provide a comprehensive resource ranging from detailed explanations to molecular docking strategies for naturally occurring compounds with anti-inflammatory activity [165,166]. Recently, the creation of anti-inflammatory substances based on plant SMs, such as polyphenols, terpenes, fatty acids and many other bioactive components, have demonstrated noteworthy efficacy. In particular, Aswad et al. [167] reported that many plant SMs such as moupinamide, capsaicin and hypaphorine derived from *Zanthoxylum beecheyanum*, chili pepper and *Erythrina velutina*, respectively, can be used as new promising anti-inflammatory drugs.

#### 5.3.4. Anticancer

Several plant SMs have been reported to possess potential anticancer activity due to their capacity to prevent oxidative stress and inflammation that causes damage to DNA, which in turn leads to carcinogenesis [168]. Natural products, such as irinotecan, vincristine, vinblastine, etoposide and paclitaxel from plants; actinomycin D and mitomycin C from bacteria; and marine-derived bleomycin, are widely used in various cancer therapies [169]. Moreover, fruits and vegetables containing vitamins, minerals, folate, plant sterols, carotenoids and phytochemicals, such as flavonoids and polyphenols, are associated with reduced cancer mortality [170]. Herbs and spices such as ginger, capsicum, curcumin, clove, rosemary, sage, oregano and cinnamon are very rich in antioxidants due to the high content of phenolic compounds, and they have been shown to counteract reactive oxygen species (ROS)-mediated damage in different human cancers [166].

## 6. Metabolomics: Technology Development and Experimental Approaches

Metabolomics is defined as a comprehensive quantitative and qualitative analysis of all metabolites present in a specific cell, tissue or organism. Targeted and global (or unbiased) metabolite analysis are the two main metabolomics methodologies. The chemical characterization for plant metabolites, particularly SMs, is a subset of analytic techniques such as gas chromatography–mass spectrometry (GC–MS) and liquid chromatography–mass spectrometry (LC–MS), together with an estimate of quantity. Various other techniques, including thin layer chromatography (TLC), Fourier transform infrared spectroscopy (FT-IR), Raman spectroscopy and nuclear magnetic resonance (NMR) are also part of the metabolite analysis [171,172]. One-dimensional (1D) NMR spectrometry has shown its utility for high-throughput analysis and classification of similar chemical groups of samples; however, the large numbers of overlapping peaks generated by this method may hinder the accurate identification of specific metabolites. Recently, by replacing the 1D ^1^HNMR spectroscopic technology, a two-dimensional (2D) ^1^H–^13^C NMR strategy for the analysis of metabolites as multivariate statistical objects has been developed [173]. The new advanced techniques, including various forms of liquid chromatography with NMR, such as HPLC–SPENMR, have improved the sensitivity of NMR analyses and are capable of characterizing both the high- and low-abundance metabolites in complex crude plant extracts [174]. Mass spectroscopy (MS) is currently the most widely applied technology in metabolomics. Among a variety of MS techniques, GC–MS has been long used in metabolite profiling of human body fluids or plant extracts [175,176].

## 7. Conclusions

Plants are a significant source of NPs that are used in various fields, including agro-pharmaceuticals, plant protection, plant growth-promoting, food preservatives, novel drugs, etc. Additionally, plant metabolites are considered a significant source of food additives due to their potent antioxidant action. Recently, plant SMs have gained considerable economic attention, which has enhanced their production and led to the identification of potential new biological applications for them. The current review study focused mainly on a number of biological and pharmacological characteristics of plant bioactive metabolites. In particular, this review has underlined the following points of plant metabolites: historical overview, nutritional, cosmetic, medical and agricultural uses, as well as their impact on plant protection and growth-promoting. The classification of plant metabolites was also considered in this review, focusing mainly on the main common SMs such as terpenoids, phenolic compounds (flavonoids) and nitrogen-containing compounds (alkaloids). Furthermore, the biological activity of plant SMs, either antimicrobial and/or antioxidant, was also discussed. Different pharmacological properties of plant SMs, such as antibiotic, antiviral, anti-inflammatory, anticancer and hepatoprotective activities, were also discussed. In conclusion, we hope that the information reported in this review will be helpful for whom are interested in studying plant NPs particularly secondary metabolites.

## Figures and Tables

**Table 1:** Some common plant terpenoids.

Compound	Plant Source	Activity	References
Azadirachtin	Seeds of the neem tree (*Azadirachta indica*)	Insecticidal. Azadirachtin considered the active component in many pesticides, including TreeAzin, AzaMax, AzaSol and Terramera Cirkil.	[90]
Bornyl acetate (ester)	Many plant EOs such as cedars, hemlocks, pines and spruces.	Antibacterial	[91]
Camphene (bicyclic monoterpene),	Minor constituent of many aromatic EOs such as camphor, citronella and ginger EOs. Major constituents of thymus, oregano and sage EOs.	Antibacterial and anticancer. Used in pulmonary disease.	[92,93]
Limonene (cyclic momoterpene)	Main component of citrus fruit peels oil	Antibacterial, antifungal and food preservation.	[94,97]
α-Pinene (unsaturated bicyclic monoterpene)	Many coniferous trees.	Antimicrobial, antioxidant, anti-inflammatory and anti-carcinogenic.	[96,97,98]
Saponins (triterpene glycosides)	*Allium* species (onion, garlic), asparagus, oats, spinach, sugar beet, tea and sweet potato.	Hypolipidemic, it can reduce cholesterol and lipoprotein levels. Anti-diabetic and anti-carcinogenic.	[99,100]
3-Carene (bicyclic monoterpene)	Rosemary, citrus, basil, cedar, pine and cannabis	Antimicrobial effect against some food-borne pathogenic bacteria. Used also as food additive.	[101,102]

**Table 3:** Some common plant alkaloids.

Compound	Plant Source	Activity	References
Morphine	*Papaver somniferum*	Analgesic	[44,120,121]
Quinine	*Remijia* sp.	Antimalarial (Antimicobial)	[122]
Ephedrine	*Ephedra* sp.	Antiasthma (Anti-inflammatory)	[124]
Homoharringtonine	*Cephalotaxus fortunei*	Anticancer activity	[126,127]
Vincamine	*Vinca minor*	Vasodilatory	[128]
Chelerythrine	*Chelidonium majus*	Antibacterial (Antimicrobial)	[130,131]
Piperine	Fruits of *Piper longum* and *Piper officinarum*	Antihyperglycemic	[132]

## Data Availability

Not applicable.
